# Alveolar Ridge Preservation Using Leukocyte and Platelet-Rich Fibrin: A Report of a Case

**DOI:** 10.1155/2011/345048

**Published:** 2011-07-03

**Authors:** Mogammad Thabit Peck, Johan Marnewick, Lawrence Stephen

**Affiliations:** Department of Oral Medicine and Periodontology, Dental Faculty, University of the Western Cape, Tygerberg Campus, Private Bag X7501, Bellville 7535, South Africa

## Abstract

In order for a dental implant to be restored optimally, it must be placed in an ideal anatomic position. However, this is not always possible, since physiological wound healing after tooth removal, often results in hard and soft tissue changes which ultimately compromises ideal implant placement. With the aim of minimising the need for tissue augmentation, several alveolar ridge preservation (ARP) techniques have been developed. These often require the use of grafting material and therefore increase the risk of disease transmission. Leukocyte and platelet-rich fibrin (L-PRF) is a newly developed platelet concentrate that is prepared from the patient's own blood. Clinical research has indicated that it improves wound healing and stimulates bone formation. We present a case where L-PRF was successfully used in an ARP procedure to facilitate implant placement in a compromised extraction socket.

## 1. Introduction

In order for a dental implant to be restored optimally, it must be placed in an ideal anatomic position. However, this is not always possible, since physiological wound healing following either tooth extraction, trauma, or pathology, often results in a deficiency of both hard and soft tissue. Unless augmentation procedures are carried out, placing an implant in these tissue-deficient sites would ultimately compromise the functional and aesthetic results [1]. Although several different augmentation procedures have been developed, many of them are associated with a number of disadvantages such as increased overall cost, the requirement for a second surgical site, and the use of animal derived products [2]. 

With the aim of minimizing the need for tissue augmentation, several authors have proposed techniques to preserve the anatomy of the alveolar ridge after tooth extraction. These procedures have collectively been termed alveolar ridge preservation (ARP) or socket preservation [2]. Several different ARP techniques exist, most of which include the use of a foreign graft materials. Because ARP is a relatively new procedure, no long-term studies regarding the technique have been published, and even though several case reports have been presented, there is no evidence to support the superiority of one technique over the other. 

Recently, Choukroun introduced leukocyte and platelet-rich fibrin (L-PRF), a second-generation platelet concentrate that improves healing of the both hard and soft tissues [3]. We present a case where L-PRF was used in an ARP procedure to limit ridge resorption after tooth extraction, in order to maximise the tissue available for ideal implant placement. 

## 2. Case Presentation

**Figure 1:**
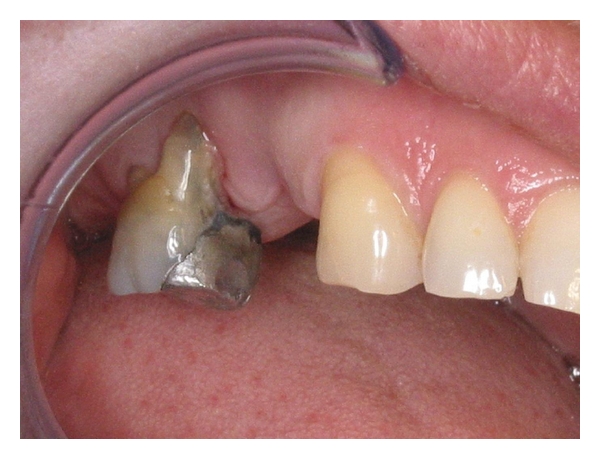
Initial presentation.

**Figure 2:**
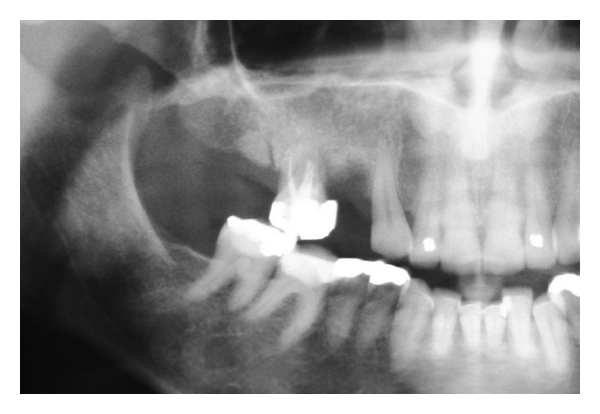
Radiograph shows hopeless upper molar with retained roots both mesial and distal to the tooth.

A 43-year-old healthy female presented for the restoration of her dentition in the upper right jaw. Upon clinical examination, it was noted that several posterior teeth were missing from the first quadrant and that the only remaining multirooted tooth (the upper right first molar) was severely periodontally compromised (Figure 1). The radiographic examination revealed the presence of unextracted roots in the areas immediately mesial and distal to the remaining molar (Figure 2). Based on the poor prognosis of the molar as well as the presence of the unextracted roots, a treatment plan that involved the extraction of the remaining tooth and roots, and subsequently replacing them with an implant-supported prosthesis, was deemed the best long-term restorative solution. In order to maximise the amount of available bone for implant placement, an ARP procedure was indicated at the time of extraction.

## 3. Initial Visit

**Figure 3:**
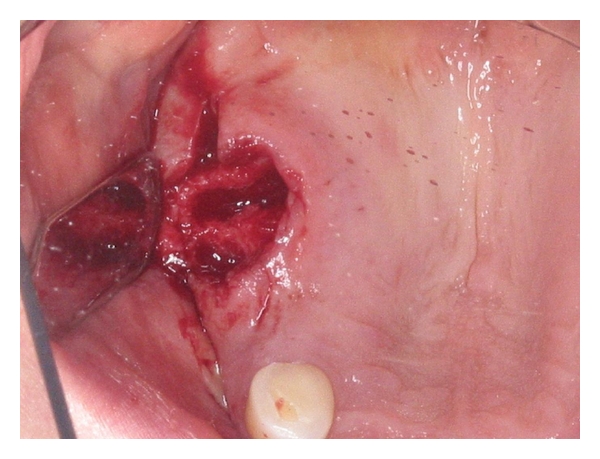
Extraction site immediately after tooth removal.

**Figure 4:**
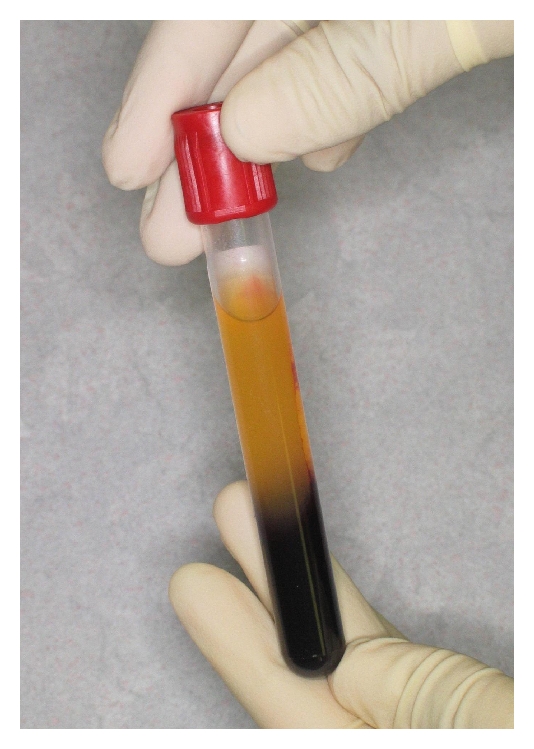
Formation of L-PRF.

**Figure 5:**
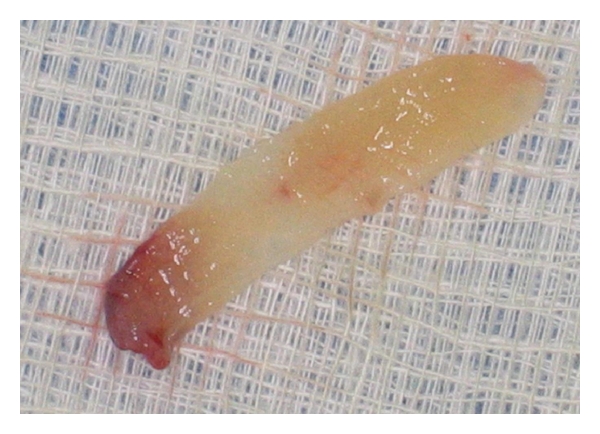
L-PRF membrane.

After local anaesthesia had been obtained, the upper right molar together with the residual tooth roots were extracted atraumatically by using a 5 mm dental luxator (Dentsply Ltd., Surrey, United Kingdom). The remaining tooth sockets were curetted and all granulation tissue and socket debris were removed (Figure 3). At the same time, 30 mL of blood was drawn from the antecubital fossa of the patient into three separate blood collecting tubes (Vacuette with Z Serum Clot Activator, Greiner Bio One International AG, Germany). These were then immediately centrifuged at 400 g for 12 minutes, using a standard tabletop laboratory centrifuge (PLC-03, Hi-care International, Taiwan). Using this method, the blood in the tubes separated into three visible layers, that is, a red blood cell layer (RBC) that occupied the lower most part of the tube, a cell-free layer that occupied the uppermost part of the tube, and an L-PRF layer that was located between the two (Figure 4). For each tube, the L-PRF layer was removed, and compressed between saline soaked sterile gauze to form an “L-PRF membrane” (Figure 5). A total of three L-PRF membranes were formed and inserted into the extraction socket site. These were then stabilised using 4–0 braided resorbable sutures (Clinisut, Port Elizabeth, South Africa) that were sutured over the wound site. Oral analgesics and a chlorhexidine 0.2% mouth rinse was prescribed during the healing period, and the patient was followed up two weeks later.

## 4. Follow-Up Visit

**Figure 6:**
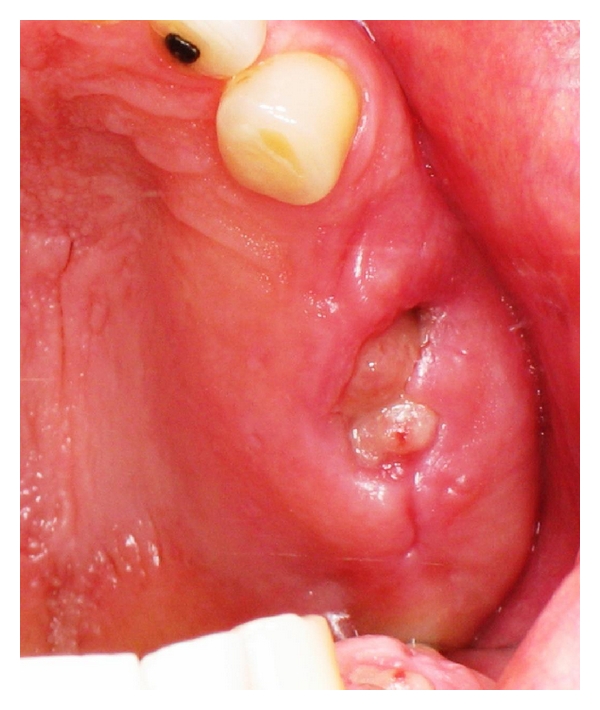
Extraction site healing 1 week after tooth removal (note the visibility of the L-PRF membrane).

On the follow-up visit, the extraction site showed signs of healing with no evidence of residual inflammation. The site was free of infection and the L-PRF membrane was still clearly visible (Figure 6). Even though it remained exposed to the oral environment, there were no signs of membrane disintegration or infection. The patient also reported minimal pain during the postoperative period. Because of the positive response to treatment, she was scheduled for implant placement four weeks later.

## 5. Implant Placement

**Figure 7:**
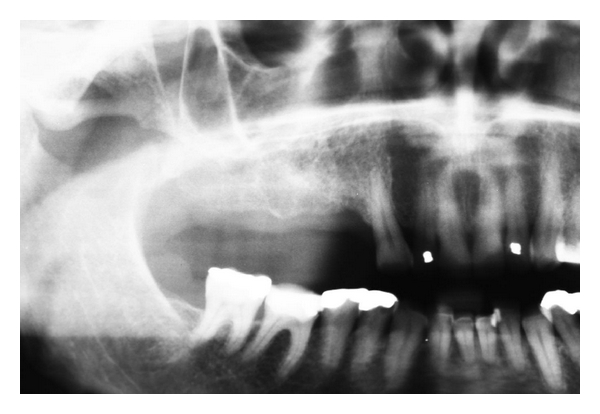
Radiograph showing new bone formation.

**Figure 8:**
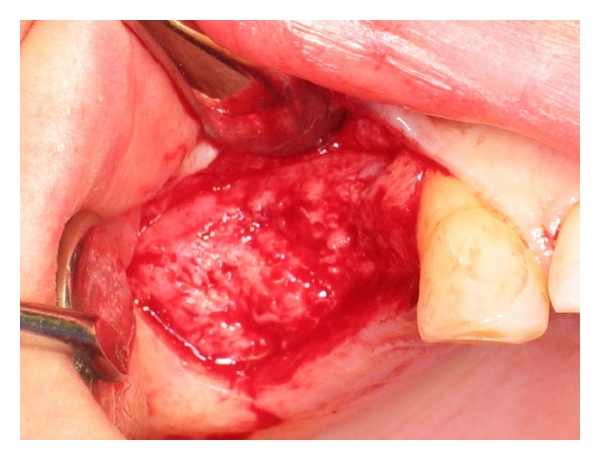
Flap reflection.

Implant placement was carried out 6 weeks after tooth extraction. A radiograph taken prior to implant placement confirmed new bone formation in the extraction area (Figure 7). Upon surgical flap reflection, the underlying alveolar ridge was clearly visible. The ridge had retained its morphology with no signs of bone resorption or of the residual socket (Figure 8). At implant insertion, the quality of the newly formed bone was such that it allowed for the implant to be inserted at an insertion torque of more than 35 Ncm.

## 6. Prosthetic Management

Eight weeks after implant placement, the implant was restored with a cement retained crown and has since then remained in function without any complications. 

**Figure 9:**
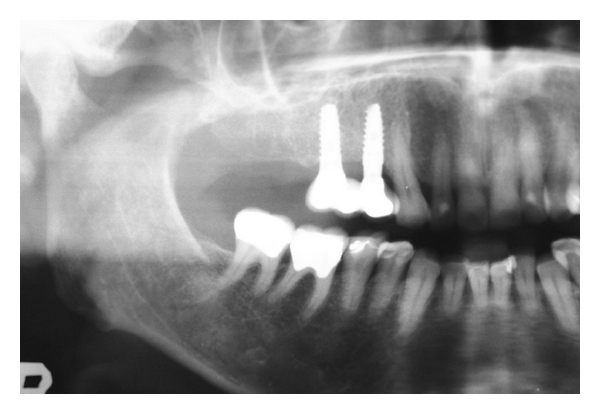
Radiograph showing stable peri-implant bone 3 months after restoration.

At the 3 month follow-up after the restorative treatment had been completed, radiographic evidence of bone maturation was present at the peri-implant sites (Figure 9).

## 7. Discussion

The healing of an extraction socket is characterised by both internal and external changes that ultimately affect the shape of the alveolar ridge [2]. Studies indicate that during healing, bone does not regenerate to the level of bone crest or to the level of the neighbouring teeth, and therefore 100% socket fill does not occur. Using an animal model, Araujo and Lindhe showed that in the first 8 weeks following extraction, there is marked osteoclastic activity, resulting in the resorption of the facial and lingual bone walls, especially in the crestal region [4]. They also noted that bone resorption was greater on the facial wall and that any loss of ridge height was accompanied by a horizontal losson both facial and lingual walls of the extraction site.

Alveolar ridge preservation is a relatively new surgical procedure aimed at retaining maximum bone and soft tissue after a tooth has been removed [2]. By maintaining the original ridge morphology, there will be a minimal need for augmentation procedures thereby allowing the resultant restoration to be placed in an aesthetically and functionally ideal position. 

During the last decade several different ARP techniques have been developed, most of which include the use of a graft material that is placed into the extraction socket [2]. This increases the treatment cost as well as increases the risk of disease transmission. Studies also indicate that in many cases, the graft material is not totally incorporated into the newly formed bone and when compared to sites without graft material, they show less vital bone formation. In some cases ARP requires the use of collagen membranes. In these cases a 25% membrane exposure rate has been reported, and this directly affects the amount of bone fill that takes place within the socket [2]. 

Leukocyte and platelet-rich fibrin (L-PRF) was first described by Choukroun as cited by Dohan et al. 2006 [5]. It is considered a second-generation platelet concentrate and has been used in various surgical procedures in an attempt to enhance wound healing. It is prepared from the patient's own blood thereby eliminating the possibility of disease transmission or foreign body reactions. 

The preparation technique of L-PRF is simple and requires no special equipment. Blood is drawn into standard glass/silica coated blood collection tubes and centrifuged at a predetermined speed to ensure cell separation. No anti-coagulants are used during the procedure and natural coagulation can therefore take place. This unique preparation technique allows L-PRF to trap at least 95% of the platelets of the collected blood into a fibrin mesh [6]. The fibrin mesh can then be easily manipulated into a membrane that allows it to be transferred to any surgical site. Here, high concentrations of the collected platelets allow for the slow release of growth factors (GFs) from the platelet granules [7]. These GFs include vascular endothelium growth factor (VEGF), platelet-derived growth factor (PDGF), fibroblast growth factor (FGF), epidermal growth factor (EGF), hepatocyte growth factor (HGF), insulin-like growth factor (IGF), platelet-derived growth factor (PDGF), and transforming-growth factor-beta (TGF-beta). All of these play a role in replacing lost tissue, resurfacing of the wound, and restoring vascular integrity. Compared to other platelet concentrates, L-PRF releases these factors at a sustained rate over a longer period, thereby optimising wound healing [8]. Recently, L-PRF has also been shown to stimulate the growth of osteoblasts and periodontal ligament cells, both of which are significant for the regeneration of periodontal defects [6, 8–11]. 

Because of the *in vitro* efficacy of L-PRF, several clinical studies have been carried out to determine its clinical potential. Currently, L-PRF has been successfully tested in a number of procedures including maxillofacial surgery, periodontal surgery, and implantology [9]. Mazor et al. successfully used L-PRF as the only grafting material in a series of sinus augmentation procedures [10]. With this technique Mazor et al. were able to demonstrate that L-PRF could stimulate new bone formation in areas that were previously deficient of the amount of bone required for implant placement [10]. In a similar 6-year follow-up study, Simonpeiri et al. were able to demonstrate that using L-PRF as a sole grafting agent was a viable long-term option in sinus augmentation procedures [11]. 

L-PRF has also been used successfully to treat periodontal defects. *In vitro* studies have confirmed that L-PRF selectively stimulates the growth of osteoblasts and gingival cells [12]. In a series of clinical trials conducted by Pradeep and Sharma it was shown that L-PRF could be used as a guided-tissue-regeneration (GTR) membrane to affect periodontal regeneration in 3-wall bony defects and degree II furcation lesions [9, 13]. Del Corso et al. published several case reports showing the successful use of L-PRF membranes in the management of both single and multiple gingival recession defects [14]. The clinical results were maintained successfully for at least one year. Ramakrishnan et al. confirmed this observation and showed that L-PRF could be used for root coverage procedures [15].

## 8. Conclusion

In the above case report, we demonstrated the successful use of L-PRF in an ARP procedure. The biomaterial acts by releasing high-concentration growth factors to the wound site, thereby stimulating healing and new bone formation [16]. Unlike other ARP procedures, the use of L-PRF is a simple method that requires minimal cost and reduces the need for specialised grafting material. Because it is a completely autologous product, the risk of disease transmission and graft rejection is negated. Further long-term research is required to determine whether ARP procedures would benefit from the use of L-PRF.

## References

[1] Chiapasco M, Casentini P, Zaniboni M (2009). Bone augmentation procedures in implant dentistry. *The International Journal of Oral & Maxillofacial Implants*.

[2] Darby I, Chen ST, Buser D (2009). Ridge preservation techniques for implant therapy. *The International Journal of Oral & Maxillofacial Implants*.

[3] Choukroun J, Diss A, Simonpieri A (2006). Platelet-rich fibrin (PRF): a second-generation platelet concentrate. Part V: histologic evaluations of PRF effects on bone allograft maturation in sinus lift. *Oral Surgery, Oral Medicine, Oral Pathology, Oral Radiology and Endodontology*.

[4] Araújo MG, Lindhe J (2009). Ridge alterations following tooth extraction with and without flap elevation: an experimental study in the dog. *Clinical Oral Implants Research*.

[5] Dohan DM, Choukroun J, Diss A (2006). Platelet-rich fibrin (PRF): a second-generation platelet concentrate. Part II: platelet-related biologic features. *Oral Surgery, Oral Medicine, Oral Pathology, Oral Radiology and Endodontology*.

[6] Ehrenfest DMD, Del Corso M, Diss A, Mouhyi J, Charrier JB (2010). Three-dimensional architecture and cell composition of a Choukroun’s platelet-rich fibrin clot and membrane. *Journal of Periodontology*.

[7] Kang YH, Jeon SH, Park JY (2011). Platelet-rich fibrin (PRF) is a bioscaffold and reservoir of growth factors for tissue regeneration. *Tissue Engineering*.

[8] Blair P, Flaumenhaft R (2009). Platelet [alpha]-granules: basic biology and clinical correlates. *Blood Reviews*.

[9] Pradeep AR, Sharma A Autologous platelet rich fibrin in the treatment of mandibular degree Ii furcation defects: a randomized clinical trial.

[10] Mazor Z, Horowitz RA, Del Corso M, Prasad HS, Rohrer MD, Dohan Ehrenfest DM (2009). Sinus floor augmentation with simultaneous implant placement using Choukroun’s platelet-rich fibrin as the sole grafting material: a radiologic and histologic study at 6 months. *Journal of Periodontology*.

[11] Simonpieri A, Choukroun J, Del Corso M, Sammartino G, Dohan Ehrenfest DM (2011). Simultaneous sinus-lift and implantation using microthreaded implants and leukocyte- and platelet-rich fibrin as sole grafting material: a six-year experience. *Implant Dentistry*.

[12] Effects of platelet-rich fibrin on human periodontally related cells. http://iadr.confex.com/iadr/2008Toronto/techprogram/abstract_104888.htm.

[13] Pradeep AR, Sharma A Treatment of 3-wall intrabony defects in chronic periodontitis subjects with autologous platelet rich fibrin—a randomized controlled clinical trial.

[14] Del Corso M, Sammartino G, Dohan Ehrenfest DM (2009). Re: clinical evaluation of a modified coronally advanced flap alone or in combination with a platelet-rich fibrin membrane for the treatment of adjacent multiple gingival recessions: a 6-month study. *Journal of Periodontology*.

[15] Ramakrishnan T, Vijayalakshmi R, Pameela E, Anilkumar K, Geetha A, Umasudhakar (2009). Platelet-rich-fibrin: a novel root coverage approach. *Journal of Indian Society of Periodontology*.

[16] Kang YH, Jeon SH, Park JY (2011). Platelet-rich fibrin is a Bioscaffold and reservoir of growth factors for tissue regeneration. *Tissue Engineering*.

